# Early detection and lesion visualization of pear leaf anthracnose based on multi-source feature fusion of hyperspectral imaging

**DOI:** 10.3389/fpls.2024.1461855

**Published:** 2024-10-08

**Authors:** Yingying Zhang, Xue Li, Meiqing Wang, Tao Xu, Kai Huang, Yuanhao Sun, Quanchun Yuan, Xiaohui Lei, Yannan Qi, Xiaolan Lv

**Affiliations:** ^1^ School of Agricultural Engineering, Jiangsu University, Zhenjiang, China; ^2^ Institute of Agricultural Facilities and Equipment, Jiangsu Academy of Agricultural Sciences, Nanjing, Jiangsu, China; ^3^ Key Laboratory of Horticultural Equipment, Ministry of Agriculture and Rural Affairs, Nanjing, China; ^4^ Department of Environmental Systems Science, Institute of Agricultural Sciences, Eidgenössische Technische Hochschule (ETH) Zürich, Zürich, Switzerland

**Keywords:** hyperspectral imaging, pear leaves, anthracnose, multi-source features, classification model, visualization

## Abstract

Pear anthracnose, caused by Colletotrichum bacteria, is a severe infectious disease that significantly impacts the growth, development, and fruit yield of pear trees. Early detection of pear anthracnose before symptoms manifest is of great importance in preventing its spread and minimizing economic losses. This study utilized hyperspectral imaging (HSI) technology to investigate early detection of pear anthracnose through spectral features, vegetation indices (VIs), and texture features (TFs). Healthy and diseased pear leaves aged 1 to 5 days were selected as subjects for capturing hyperspectral images at various stages of health and disease. Characteristic wavelengths (OWs1 and OWs2) were extracted using the Successive Projection Algorithm (SPA) and Competitive Adaptive Reweighted Sampling (CARS) algorithm. Significant VIs were identified using the Random Forest (RF) algorithm, while effective TFs were derived from the Gray Level Co-occurrence Matrix (GLCM). A classification model for pear leaf early anthracnose disease was constructed by integrating different features using three machine learning algorithms: Support Vector Machine (SVM), Extreme Learning Machine (ELM), and Back Propagation Neural Network (BPNN). The results showed that: the classification identification model constructed based on the feature fusion performed better than that of single feature, with the OWs2-VIs-TFs-BPNN model achieving a highest accuracy of 98.61% in detection and identification of pear leaf early anthracnose disease. Additionally, to intuitively and effectively monitor the progression and severity of anthracnose in pear leaves, the visualization of anthracnose lesions was achieved using Successive Maximum Angle Convex Cone (SMACC) and Spectral Information Divergence (SID) techniques. According to our research results, the fusion of multi-source features based on hyperspectral imaging can be a reliable method to detect early asymptomatic infection of pear leaf anthracnose, and provide scientific theoretical support for early warning and prevention of pear leaf diseases.

## 1 Introduction

Pear trees are highly susceptible to pathogenic infections throughout their growth cycle, with anthracnose fungus being the most significant threat (Huang et al., 2015; Zhang and Xie, 2019). This fungus is highly contagious and presents a severe risk to pear leaves. If anthracnose is not detected and treated promptly, it can lead to widespread outbreaks, significantly reducing fruit quality and production, ultimately resulting in substantial economic losses (Luo, 2018). Therefore, early detection of pear leaf anthracnose is important for timely treatment, which helps ensure healthy harvests and keeps pear farming profitable.

In the current landscape of detecting pear leaf anthracnose, primary methodologies include manual visual inspection, thermal infrared imaging, machine vision, and spectral analysis. Despite their effectiveness, each approach has its limitations. Manual visual inspection, for instance, is highly subjective and often results in delayed detection of disease progression, affecting timely intervention (Hu et al., 2022; Zhang et al., 2023). Thermal infrared imaging, while useful, is affected by ambient temperature and humidity, which can compromise its accuracy (Zhu et al., 2018; Rippa et al., 2024; Masri et al., 2017). Machine vision, on the other hand, is restricted to external characteristics and fails to provide insights into internal leaf conditions (Chen et al., 2018; Palei et al., 2023; Kim et al., 2020). Spectral analysis, although capable of internal assessment, lacks the ability to detect external features (Zahir et al., 2022; Li et al., 2013). Pathogenic infections in plants result in changes to both internal physiological characteristics and external texture features, producing distinct spectral signatures divergent from healthy states. To detect diseases at an early stage, when symptoms are mild, it is crucial to monitor changes in both the internal and external aspects of the plant. Hyperspectral imaging (HSI) technology effectively combines the strengths of machine vision and spectral analysis, enabling the simultaneous collection of both spectral and visual data. This comprehensive integration can capture changes in both the internal and external aspects of plants, making the technology highly sensitive to subtle variations and crucial for the early detection of plant diseases (Zhang et al., 2021; Xie et al., 2018).

In recent years, numerous researchers have explored the early detection of plant diseases using spectral characteristics based on HSI technology. Kong et al. (2018) achieved early detection of oilseed rape leaf spot disease using a combination of HSI technology, chemometrics, and various machine learning algorithms. Hernández et al. (2024) utilized spectral features as input variables to establish an early detection model for grapevine downy mildew using Convolutional Neural Networks (CNN), with the accuracy of 99%. Huang et al. (2023) devised an SG-SVM model for early detection of sugarcane leaf spots and rust diseases. Tang et al. (2023) amalgamated the global and local spectral features, employing Support Vector Machine (SVM) to establish an early detection model for citrus anthracnose, achieving an average detection accuracy of 91.97%. When plants are infected with pathogens, various vegetation indices (VIs) such as internal moisture, pigment content, and structure will change as well, which usually have a close relationship with spectra count of different specific wavelengths, serving as features for early detection of plant diseases (Guo et al., 2024). Abdulridha et al. (2020) conducted detection of various stages of powdery mildew in pumpkins, discovering that Water Index (WI) and Photochemical Reflectance Index (PRI) accurately classified asymptomatic, early, and advanced stages of powdery mildew under laboratory conditions. Su et al. (2024) utilized six VIs, including Normalized Pigment Chlorophyll Index (NPCI), Water Index (WI), Chlorophyll Index Rededge (CIrededge), Green Atmospherically Resistant Index (GARI), Normalized Difference Vegetation Index (NDVI), and Chlorophyll Index Green (CIgreen), to detect various stages of stripe rust in wheat. The results demonstrated the effectiveness of these indices in dynamically characterizing the severity of stripe rust infection. Texture, as a crucial feature, describes the spatial distribution of brightness among adjacent pixels and stands. After plant infection with pathogens, the leaves will exhibit morphological changes such as chlorophyll loss, deformation, curling, wilting, etc. Therefore, several studies have attempted to identify plant diseases using TFs extracted from images of the plants. Xie et al. (2015) proposed detection models for early blight and late blight in tomato leaves based on 8 texture features, combined with an Extreme Learning Machine (ELM) classifier. The accuracy of the models ranged from 69.9% to 71.8%. Zhu et al. (2017) extracted TFs using the Gray-Level Co-occurrence Matrix (GLCM) and constructed various machine learning models to identify healthy and diseased tobacco leaves at different stages. Among them, the Back Propagation Neural Network (BPNN) classifier achieved an impressive accuracy of up to 93.33% in the classification task. The aforementioned studies demonstrated the feasibility of utilizing TFs for early detection of plant diseases. Obviously, present research is primarily relied on HSI technology, utilizing a single feature combined with image processing techniques to achieve early detection of plant diseases. However, studies on early detection of plant diseases through the fusion of multi-source features are relatively limited.

In addition, HSI technology can also visualize the lesion, so as to intuitively and accurately understand the location of the lesion, infection mechanism and severity (Coliban et al., 2020). Guan et al. (2022) visualized the lesions on wheat leaves infected with powdery mildew 2-5 days after infection using Spectral Angle Mapper (SAM), monitoring the progression of powdery mildew over time. Pan et al. (2019) visualized the lesions of pear black spot disease using Maximum Likelihood method and Spectral Angle Mapper (SAM). Finding that SAM could obtain the occurrence rate of pear infection with black spot disease at different stages and the size of the infected area, realizing a real-time monitoring of the occurrence process of pear infection with black spot disease. Despite the crucial role of monitoring the occurrence process in disease prevention and control, there are currently no reports on the monitoring of the occurrence process of pear leaf anthracnose with a vivid and intuitive method, limiting the further development of precision pesticide application technology.

The current study aimed to use HSI technology to develop an early detection and identification model for pear leaf anthracnose. The objectives were threefold: (1) to extract and combine both internal physiological features (OWs1, OWs2, and VIs) and external texture features (TFs) to detect the disease at an early stage; (2) to evaluate different classification models (SVM, ELM, and BPNN) for optimizing detection results; (3) to visualize the progression of the disease using SMACC and SID techniques. The research will provide scientific theoretical support for the early prevention and precise treatment of pear leaf anthracnose disease.

## 2 Materials and methods

### 2.1 Experimental materials and inoculation of pathogens

Pear leaf samples were obtained from a pear orchard (32.04°N, 118.88°E) at the Jiangsu Academy of Agricultural Sciences, Jiangsu, China. The pear cultivar was ‘Sucui No.1’. In June 2023, under the guidance of plant protection personnel, healthy leaves of uniform size and relatively broad leaf surface area were randomly sampled, totaling 300 leaves. Then the leaves were immediately placed in a small cooler and transported to the laboratory for labelling and inoculation of anthracnose pathogen.

The anthracnose pathogen was provided by Plant Protection Research Institute of Jiangsu Academy of Agricultural Sciences, Jiangsu, China. During inoculation, each leaf was divided into two parts along the midrib, and a 5mm diameter fungal plug was inoculated on each side. The inoculated leaves were placed in a growth chamber for cultivation under the following conditions: temperature of 25°C, relative humidity of 85%, and simulated light for 12 hours then by 12 hours of darkness each day. The first day of pear leaf infection was defined as the day after the inoculation day.

### 2.2 Hyperspectral imaging data acquisition

**Figure 1:**
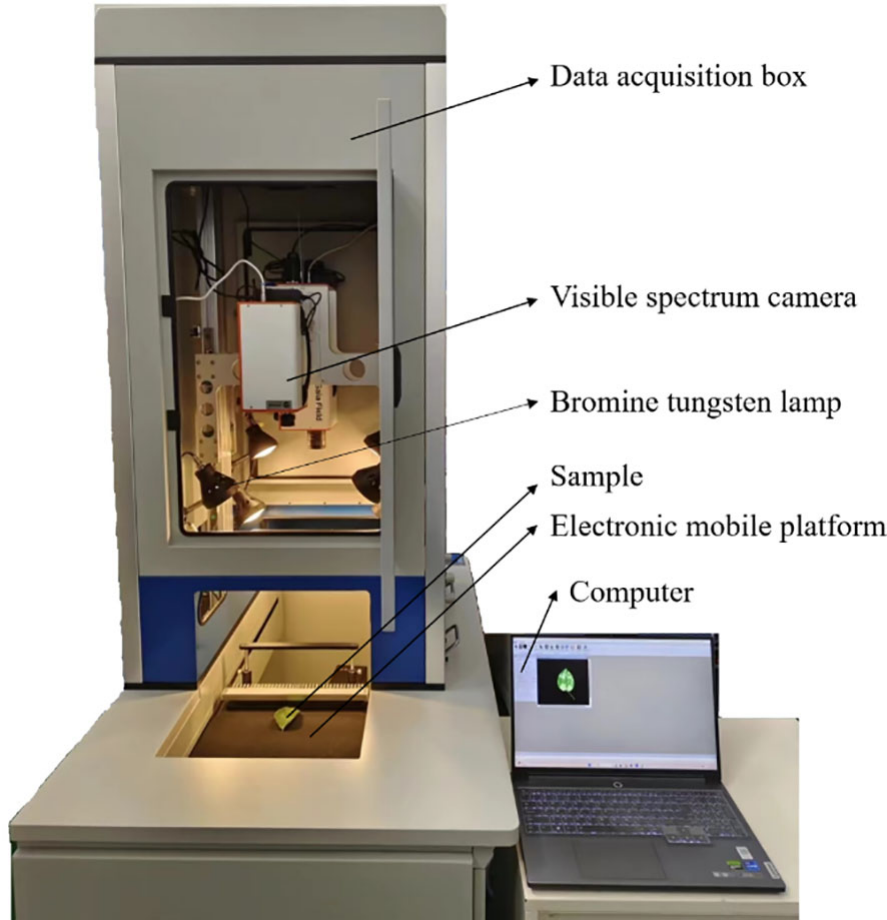
Hyperspectral image acquisition system.

**Figure 1** showed the HSI system (GaiaSorter-Dual, Jiangsu Shuangli Hesheptic Technology Co., Ltd., Jiangsu, China), which primarily consisted of two hyperspectral cameras (a visible-light camera and a near-infrared camera), a data acquisition box, and a computer. This experiment only utilized the visible-light camera, model DUALTX_IR_GE_17, with a resolution of 1392×1040 pixels, a spectral range of 380-1010 nm, a spectral resolution of 3.8 nm, and a total of 256 spectral channels. The data acquisition box comprised 8 halogen lamps (50 W each) and a motorized linear stage. During the data collection, the distance between the pear leaf surface and the camera lens was set to 55 cm, the exposure time was 9 ms, and the motor speed was 0.69 cm/s.

The infection period of the pear leaf by the anthracnose fungus was 1 day, thus the day following inoculation was considered as the first day of infection. Prior to inoculation, hyperspectral images of the pear leaves were captured for healthy controls, with each sample labeled and numbered. Subsequently, hyperspectral images were collected continuously for 5 days post-inoculation, capturing the dynamic process of the pear leaves transitioning from healthy to the onset of infection until the appearance of distinct lesions. In total, 1800 hyperspectral images were collected.

To mitigate the impact of brightness variation on data and enhance the quality and reliability of spectral data, it was essential to perform black-and-white rectification on hyperspectral data before data processing.


(1) $$R=\frac{R_{0}-B}{W-B}$$

Where, R is the corrected hyperspectral image; R_0_ is the original hyperspectral image; W is the full-white calibration image; B is the full-black calibration image.

### 2.3 Feature extraction and selection

#### 2.3.1 Region of interest extraction and selection

**Figure 2:**
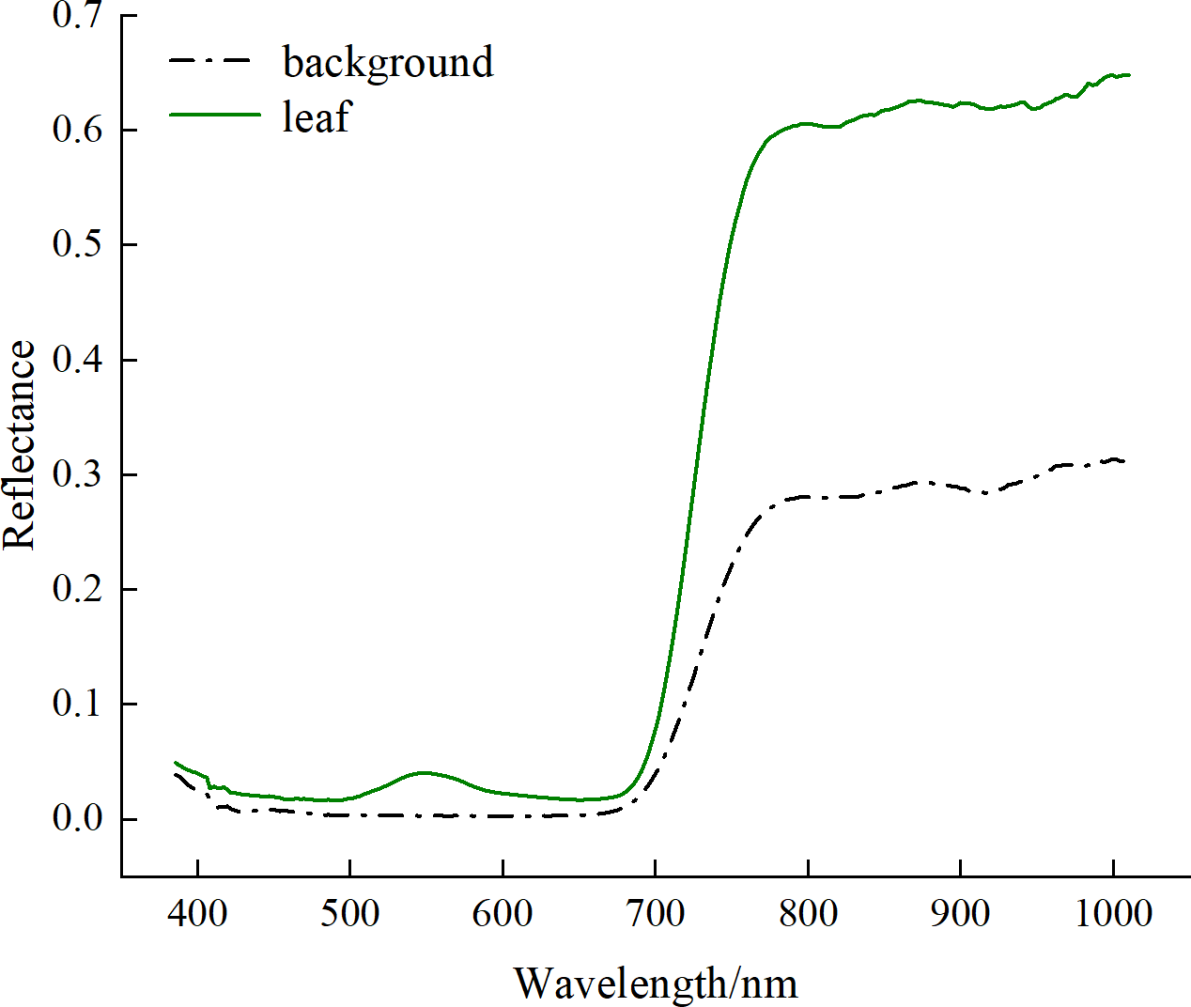
Average spectral reflectance of pear leaves and background.

In this study, the entire leaf area was considered as the region of interest (ROI) for extracting spectral information. However, the hyperspectral image collected contains both the leaf area and the background area. To extract the spectral information of the leaves, the background must first be removed. ENVI 5.3 software was used to extract the spectral reflectance of the pear leaves and the background area separately. As shown in **Figure 2**, the pear leaves exhibited a pronounced reflectance peak around 550 nm, which was significantly different from the background reflectance. Therefore, the grayscale image at the 551.2 nm band was used, and the leaves were extracted from the background using a thresholding method.

**Figure 3:**
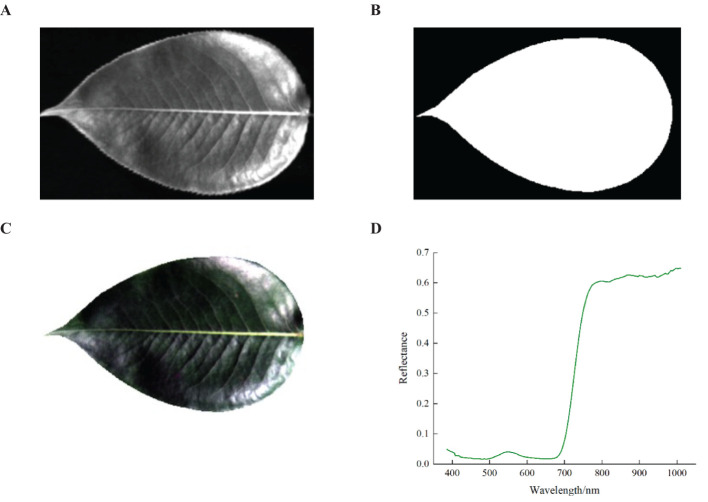
Spectral information extraction process. **(A)** Grayscale image at 551.2nm, **(B)** Binarized mask image, **(C)** Specular highlight image after background removal, **(D)** Average spectral reflectance.

Extraction Process: First, the grayscale image at 551.2 nm band was obtained using ENVI 5.3 software (**Figure 3A**). Next, the thresholding algorithm was applied to create a binary mask image, setting the leaf area pixels to 1 and the background area pixels to 0 (**Figure 3B**). Then, the mask image was then applied to all bands of the original hyperspectral image, multiplying the pixel values to obtain the hyperspectral image containing only the pear leaf area (**Figure 3C**). Finally, the average spectral reflectance curve of the leaf area was calculated by averaging the reflectance values of all pixels within the leaf region (**Figure 3D**). Additionally, the spectral reflectance values at the beginning and end of the spectral curve exhibited frequent and significant fluctuations, which were considered noise bands and lacked practical research value. Therefore, the bands with substantial noise influence were excluded, consequently 245 bands between 400 and 1000 nm were selected for subsequent analysis.

#### 2.3.2 Feature wavelength extraction and selection

Each of the captured hyperspectral images contains 245 spectral bands, with substantial inter-band correlation and redundant information, significantly affecting the speed and accuracy of subsequent data processing for modeling. Hence, reducing the dimensionality of the original spectral data and extracting optimal feature wavelengths is essential (Raghavendra et al., 2021). In this study, the Sequential Projection Algorithm (SPA) and Competitive Adaptive Reweighted Sampling (CARS) were employed for feature wavelength extraction. The SPA algorithm effectively summarizes the variable information of the majority of original spectra with a sparse subset, greatly enhancing the speed and efficiency of modeling (Araújo et al., 2001). Not only does CARS effectively eliminate non-informative variables, but it also minimizes the impact of collinear variables on the model to the greatest extent (Li et al., 2009). The extraction of feature wavelengths was carried out using the Matlab R2022b software.

#### 2.3.3 Vegetation indices extraction and selection

After being infected with anthracnose, the physiological characteristics of pear leaves change gradually, thereby altering their spectral reflectance (Pane et al., 2021). Spectral vegetation indices (VIs) are calculated using the reflectance of two or more wavelengths, enhancing the data differences between healthy and diseased samples (Sah et al., 2023; Dutta et al., 2024). This study initially selected 23 VIs related to leaf pigment, structure, and water content for the early detection of pear leaf anthracnose. The calculation formulas for each of these VIs were presented in **Table 1**.

**Table 1:** Spectral vegetation indices used in this study.

Category	Vegetation Index	Abbreviation	Equation	Reference
Pigment	Anthocyanin Reflectance Index	ARI	1/*R*_550_-1/*R*_700_	(Gitelson et al., 2005)
	Chlorophyll Index Green	CIgreen	*R*_790_/*R*_550_-1	(Gitelson et al., 2005)
	Chlorophyll Index Red	CIre	*R*_790_/*R*_720_-1	(Gitelson et al., 2005)
	Nitrogen Reflectance Index	NRI	(*R*_570_- *R*_670_)/(*R*_570_+ *R*_670_)	(Aghdam et al., 2024)
	Optimized Soil-Adjusted Vegetation Index	OSAVI	(1 + 0.16)(*R*_800_-*R*_670_)/(*R*_800_+ *R*_670_+ 0.16)	(Chandel et al., 2021)
	Photochemical Reflectance Index	PRI	(*R*_531_-*R*_570_)/(*R*_531_+ *R*_570_)	(Gamon et al., 1992)
	Plant Senescence Reflectance Index	PSRI	(*R*_660_-*R*_510_)/*R*_760_	(Penuelas et al., 1994)
	Structure Insensitive Pigment Index	SIPI	(*R*_800_-*R*_451_)/(*R*_800_+ *R*_680_)	(Penuelas et al., 1995)
	Transformed Chlorophyll Absorption Reflectance Index	TCARI	3((*R*_700_-*R*_675_)-0.2(*R*_700_-*R*_500_)/(*R*_700_+ *R*_500_))	(Merzlyak et al., 1999)
	Red Green Index	RGI	*R*_690_/*R*_550_	(Chappelle et al., 1992)
	Ratio Analysis of Reflection of Spectral Chlorophyll a	RARSa	*R*_675_/*R*_700_	(Chappelle et al., 1992)
	Ratio Analysis of Reflection of Spectral Chlorophyll b	RARSb	*R*_675_/(*R*_700_*R_650_)	(Chappelle et al., 1992)
Structure	Difference Vegetation Index	DVI	*R*_800_-*R*_680_	(Patrick et al., 2017)
	Enhanced Vegetation Index	EVI	2.5(*R*_800_-*R*_660_)/(1 + *R*_800_+ 2.4*R*_660_)	(Patrick et al., 2017)
	Green Normalized Difference Vegetation Index	GNDVI	(*R*_750_-*R*_540_+ *R*_570_)/(*R*_750_+ *R*_540_-*R*_570_)	(Patrick et al., 2017)
	Greenness Index	GI	*R*_554_/*R*_667_	(Gitelson et al., 2003)
	Normalized Difference Vegetation Index	NDVI	(*R*_800_-*R*_670_)/(*R*_800_+ *R*_670_)	(Haboudane et al., 2004)
	Ratio Vegetation Structure Index	RVSI	(*R*_651_-*R*_750_)/2-*R*_733_	(Haboudane et al., 2004)
	Triangular Vegetation Index	TVI	0.5(120(*R*_750_-*R*_550_)-200(*R*_670_-*R*_550_))	(Haboudane et al., 2004)
	Simple Ratio	SR	*R*_900_/*R*_680_	(Qiu et al., 2018)
	Ratio Vegetation Index	RVI	*R*_810_/*R*_660_	(Qiu et al., 2018)
Water content	Water Stress and Canopy Temperature	WSCT	(*R*_970_-*R*_850_)/(*R*_970_+ *R*_850_)	(Babar et al., 2006)
	Water Index	WI	*R*_900_/*R*_970_	(Penuelas et al., 1997)

To screen out the VIs that were significantly sensitive to pear leaf anthracnose, the Random Forest (RF) method was selected for feature selection. The random forest feature variable screening method has good robustness and stability. It constructs a random forest model, utilizes the bagging method and random attribute selection, evaluates the importance of each attribute, and then selects the attributes with the greatest impact on the target variable, ranking the importance of all attributes (Jaiswal and Samikannu, 2017).

#### 2.3.4 Texture feature extraction and selection

The leaf morphology of plants exhibits variation in response to the degree of anthracnose infection. Essentially, changes in pear leaf texture features (TFs) at different stages of infection can serve as a predictive indicator for the severity of anthracnose disease. In image processing, the TFs of images are typically obtained with method of Gray Level Co-occurrence Matrix (GLCM), which characterizes TFs based on pixel correlation in grayscale space and can convey directional, spatial, and range information regarding variations in image grayscale (Utaminingrum et al., 2022).

In order to improve the processing efficiency and accuracy, principal component analysis (PCA) was initially used to remove the redundant information of hyperspectral images (Licciardi and Chanussot, 2018). In this study, the first three principal components (PC1, PC2, and PC3) with a cumulative contribution of 99.5% were used as the objects for subsequent extraction of TFs. Eight TFs of Mean, Variance, Synergy, Contrast, Dissimilarity, Entropy, Second-order Moment and Correlation were selected for the followed TFs screening.

### 2.4 Model construction and performance evaluation

#### 2.4.1 Model construction

Spectral features, VIs, and TFs data obtained from the 300 pear leaf sample images daily were randomly divided in a 2:1 ratio into training and testing sets. Three machine learning algorithms, Support Vector Machine (SVM), Extreme Learning Machine (ELM), and Back Propagation Neural Network (BPNN), were employed to structure an early classification model for anthracnose disease in pear leaves. The model construction was conducted using Matlab R2022b software.

SVM is characterized by its rapid computing speed, high classification accuracy, and strong generalization ability to samples, leading to its widespread application in statistical classification and regression analysis (Cervantes et al., 2020). In this study, SVM selected the Radial Basis Function (RBF) kernel, and employed grid search method to determine the optimal values of the penalty factor c and the kernel parameter g. ELM has emerged as a highly popular machine learning algorithm. During training, it requires no adjustments; simply setting the number of neurons in the hidden layer yields a unique optimal solution (Xu et al., 2019). In this study, ELM utilized the sigmoid function as the activation function of the hidden layer, with an optimal number of hidden layer nodes obtained through cross-validation. BPNN is a type of feedforward neural network capable of easily implementing complex nonlinear mapping functions with strong generalization capabilities, finds widespread applications (Asaad and Ali, 2019). In this study, the number of nodes in the input layer of BPNN was consistent with the number of input parameters of the sample, the number of nodes in the output layer was consistent with the number of classification, the activation function of the hidden layer was tanh, the maximum number of iterations of the network was set at 1000, and the target error was 0.01. The relationship between the number Y of nodes in the hidden layer and the number I of nodes in the input layer was calculated by the following formula (Kim, 2017).


(2) Y=2I+1

#### 2.4.2 Model performance evaluation

The model performance was evaluated via accuracy, recall, and precision. “Accuracy” refers to the proportion of samples correctly predicted out of the total samples. “Recall” indicates the ratio of correctly classified samples of a certain category to the actual number of samples in that category. “Precision” denotes the ratio of correctly classified samples of a certain category to the predicted number of samples in that category (Lamba et al., 2021).


(3) $$Accuracy=\frac{\left(TP+TN\right)}{\left(TP+TN+FP+FN\right)}$$

(4) $$Recall=\frac{TP}{\left(TP+FN\right)}$$

(5) $$Precision=\frac{TP}{\left(TP+FP\right)}$$

Where, TP is the number of true positive; TN is the number of true negative; FP is the number of false positive; FN is the number of false negative.

With increasing emphasis on the accuracy of plant disease detection, accuracy was the primary evaluation metric in this study. The higher the accuracy, the better the model performance.

### 2.5 Lesion visualization

Sequential Maximum Angle Convex Cone (SMACC) is a faster and more automatic method to obtain endmember spectra. It starts with an endmember and increases in dimension. According to the angle between the end member and the existing cone, the new end member is identified. The data vector with the largest included angle with the existing cone is selected as the next endmember to expand the endmember set. Within a certain tolerance range, when all pixel vectors are within the convex cone, the algorithm terminates (Zhang, 2023; Nalawade et al., 2019).

Spectral Information Divergence (SID) is a spectral classification method. It calculates the local characteristics of spectra by spectral gradient, and then compares the overall characteristics of spectra by using information divergence to measure the degree of difference between different spectra, so as to determine their similarity or dissimilarity. By comparing the divergence of spectral information of different samples, the classification and identification of spectra can be realized (Chang, 1999).

## 3 Results

### 3.1 Spectral feature analysis and modeling

#### 3.1.1 Spectral feature analysis

The spectral reflectance data of the pear leaf samples were collected within the 400-1000 nm wavelength range. To mitigate the impact of various noise sources on the raw spectral data, a Savitzky-Golay convolution smoothing (SG) method was employed to enhance the signal-to-noise ratio and improve the subsequent data analysis (Truyols and Schoenmakers, 2006).

**Figure 4:**
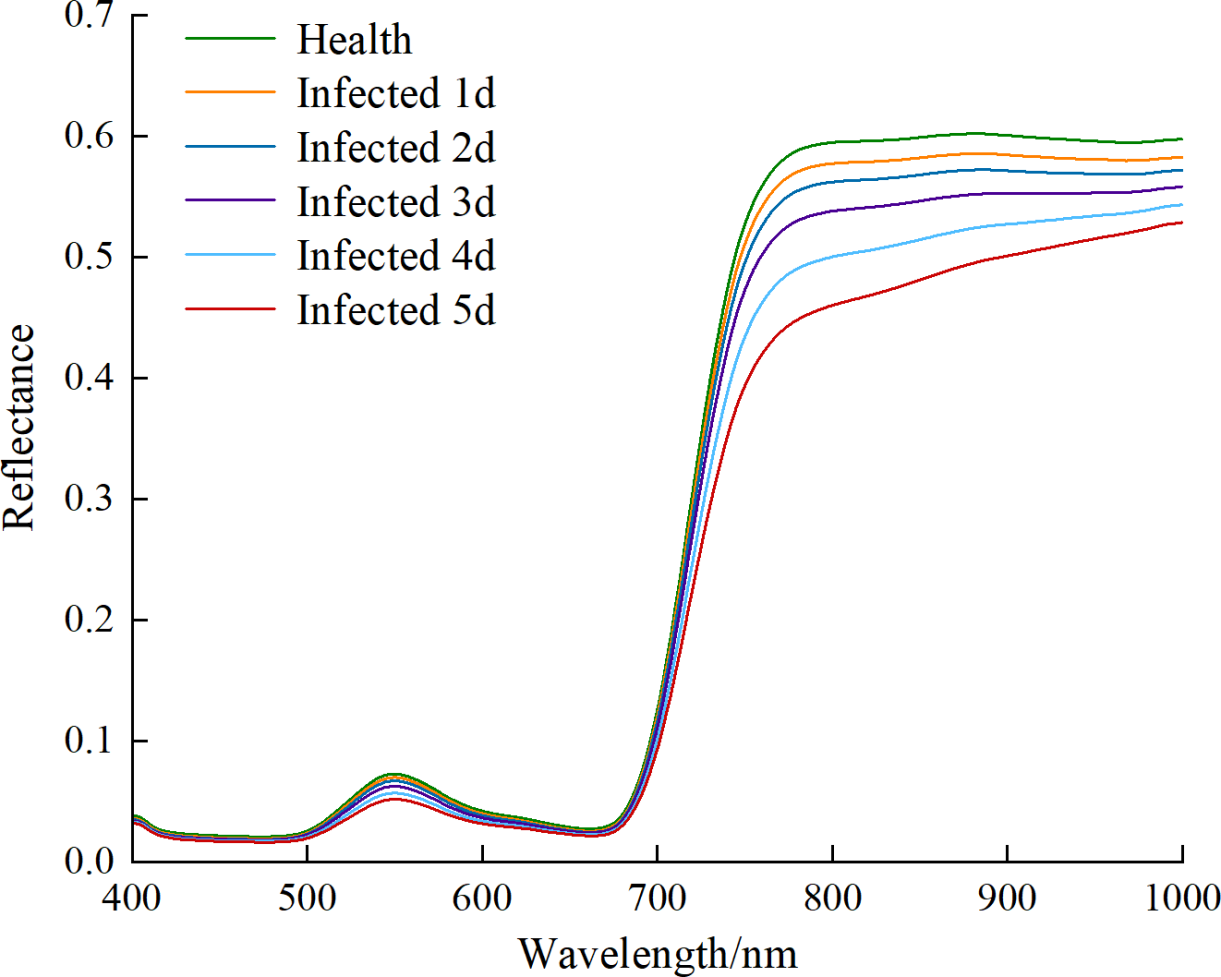
Average spectral curves of healthy and infected leaves from 1 to 5 days.

**Figure 4** depicted the average spectral reflectance curves for healthy leaves and leaves infected with anthrax from day 1 to day 5. The overall trend of the spectral curves for the healthy and infected leaves at different days was similar, exhibiting the typical spectral characteristics of green plants. At around 550 nm, there was a minimum in chlorophyll absorption, while at around 680 nm, there was a strong chlorophyll absorption, leading to the “red edge” phenomenon (Zhao et al., 2022). The spectral reflectance showed a significant increase in the 700-760 nm region, and then a more gradual change, forming a high reflectance platform after 760 nm. The peak value of the chlorophyll reflectance around 550 nm varies, which was consistent with the “loss of greenness” in the infected leaf samples. However, the difference between healthy leaves and leaves infected for 1-2 days was relatively small, likely due to the lack of visible lesions during the early stages of infection. In the 700-1000 nm spectral region, the reflectance of healthy leaves was significantly higher than that of the infected leaves as the disease progresses, which could be attributed to the damage to the leaf cell structure caused by the disease.

**Figure 5:**
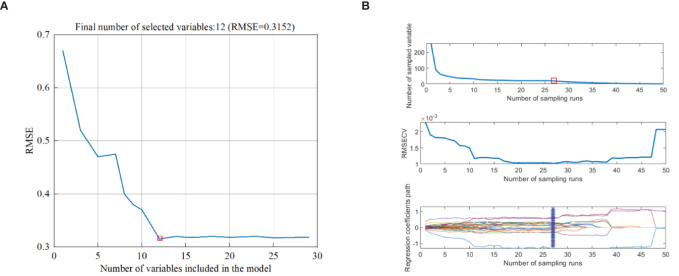
Different algorithms for extracting feature wavelengths. **(A)** Extraction of SPA Feature Wavelengths, **(B)** Extraction of CARS Feature Wavelengths.

The feature wavelength of full-band spectrum was extracted by SPA. The SPA algorithm was run through Matlab R2022b, and the average spectral curves of ROI of all samples were screened. As shown in **Figure 5A**, when RMSE reached the minimum value (RMSE=0.3152), 12 feature wavelengths were determined. The feature wavelength of the full-band spectrum was extracted by CARS. As shown in **Figure 5B**, with the increase of sampling times, the number of wavelength variables rapidly decreased until it approached 0. In the RMSECV chart, it decreased as irrelevant wavelengths were removed, followed by an upward trend. When the RMSECV value was at its lowest, it indicated that irrelevant and collinear wavelengths had been removed, and 8 feature wavelengths had been selected.

According to **Figure 5** and **Table 2**, 12 feature wavelengths (OWs1) were extracted by SPA, and 8 feature features (OWs2) by CARS, reducing 95.1% and 96.7% wavelength count, respectively, eliminating the redundant information from ineffective wavelengths and significantly improving the speed of data processing.

**Table 2:** Feature wavelengths extracted by the different algorithms.

Algorithm	Number of Feature wavelengths	Feature wavelengths/nm
SPA	12	401.6, 410.6, 449.1, 558.3, 712.8, 792.6, 825.5, 879.3, 933.8, 952.1, 999.6
CARS	8	710.6, 712.8, 792.6, 825.5, 879.3, 933.8, 952.1, 999.6

#### 3.1.2 Identification models with spectral features

In this study, full-spectrum, SPA, and CARS extracted feature wavelengths (OWs1 and OWs2) were used as input variables to construct classification models with SVM, ELM, and BPNN, respectively. The classification results of testing sets were presented in **Table 3**. The identification accuracy of the three models constructed based on the extraction wavelength of SPA and CARS exceeded that of the full-band model, signifying that the extraction of feature wavelengths not only markedly enhanced modeling speed but also augmented modeling accuracy. Among these, the OWs2-BPNN model demonstrated the most effective identification efficacy, achieving an accuracy of 95.13%.

**Table 3:** Classification results of identification models testing sets based on spectral characteristics.

Input features	Number of variables	Model type	Accuracy (%)	Recall (%)	Precision (%)
Full spectrum	245	SVM	87.50	87.48	89.13
		ELM	89.44	88.95	91.08
		BPNN	92.50	91.89	92.68
OWs1	12	SVM	88.06	89.27	89.58
		ELM	91.67	90.78	91.65
		BPNN	93.33	94.92	93.37
OWs2	8	SVM	90.00	91.23	90.82
		ELM	92.46	93.12	92.70
		BPNN	95.13	94.42	95.85

### 3.2 Vegetation indices feature analysis and modeling

#### 3.2.1 Vegetation indices feature analysis

**Figure 6:**
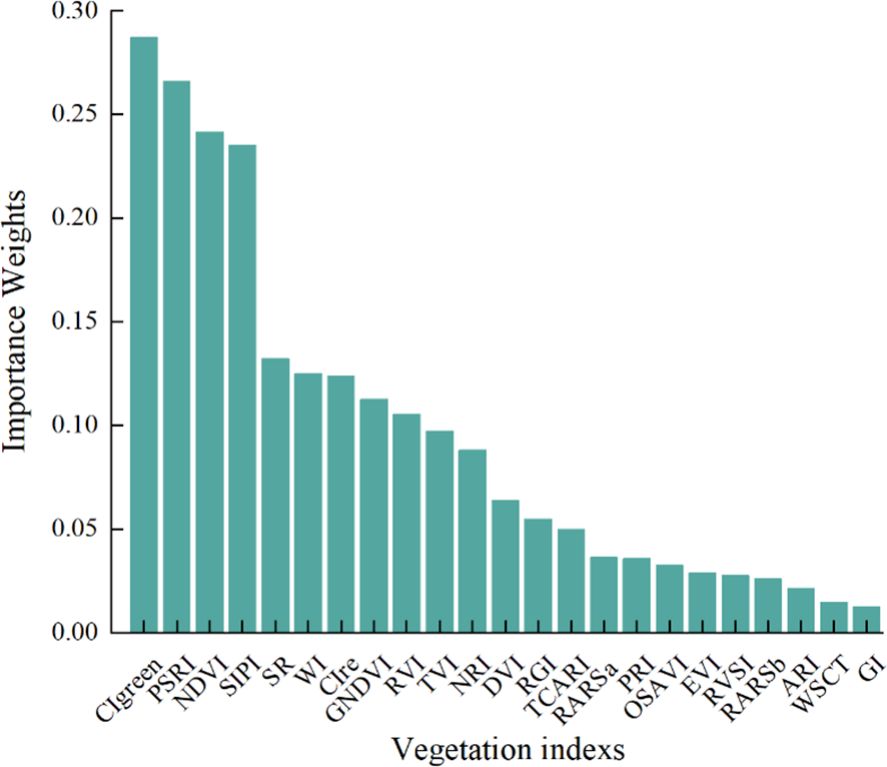
Ranking the importance weight of VIs features by random forest algorithm.

Numerous studies have demonstrated that VIs can more comprehensively explain changes in vegetation growth, and models constructed using VIs can more effectively detect plant diseases (Zhao et al., 2020). This study selected 23 VIs related to plant disease, and used RF to screen for significant VIs features sensitive to pear leaf anthracnose, ranking the importance weights of each VI feature as shown in **Figure 6**.

**Figure 7:**
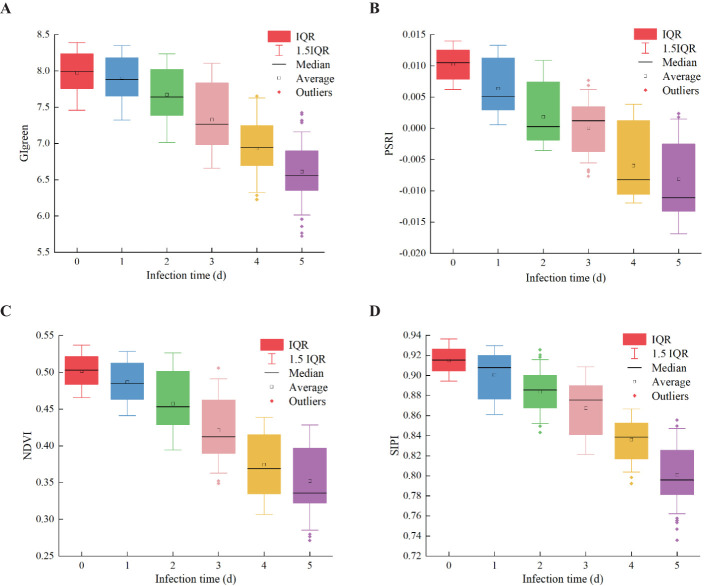
The changing trend of four VIs samples in the whole experimental stage. **(A)** CIgreen, **(B)** PSRI, **(C)** NDVI, **(D)** SIPI.

To verify the effectiveness of VIs for early detection of pear leaf anthracnose and reduce redundant information, the top 4 VIs ranked by importance weight were selected as features for early detection of pear leaf anthracnose for further analysis, they were CIgreen, PSRI, NDVI, and SIPI. The box plots of these 4 VIs in the pear leaf samples throughout the experimental stages were shown in **Figure 7**. These 4 VIs exhibited a generally monotonic change in the pear leaf samples as the infection time progressed, which was consistent with the typical pattern of disease development in pear leaves infected with anthracnose.

#### 3.2.2 Identification models with vegetation indices features

The four selected VIs screened through RF were utilized as input variables for constructing the classification models: SVM, ELM, and BPNN. The model classification results of testing sets were shown in **Table 4**. The identification accuracies of the three models were 88.06%, 92.39%, and 91.94%, respectively, indicating the effective utilization of VIs as input features for early detection of pear leaf spot disease. Among the models, the ELM model exhibited the best performance, with not only the highest accuracy but also superior recall and precision rates compared to the other two models.

**Table 4:** Classification results of identification models testing sets based on VIs features.

Input features	Number of variables	Model type	Accuracy (%)	Recall (%)	Precision (%)
VIs	4	SVM	88.06	86.78	89.18
		ELM	92.39	91.72	92.73
		BPNN	91.94	92.08	92.40

### 3.3 Texture feature analysis and modeling

#### 3.3.1 Texture feature analysis

**Figure 8:**
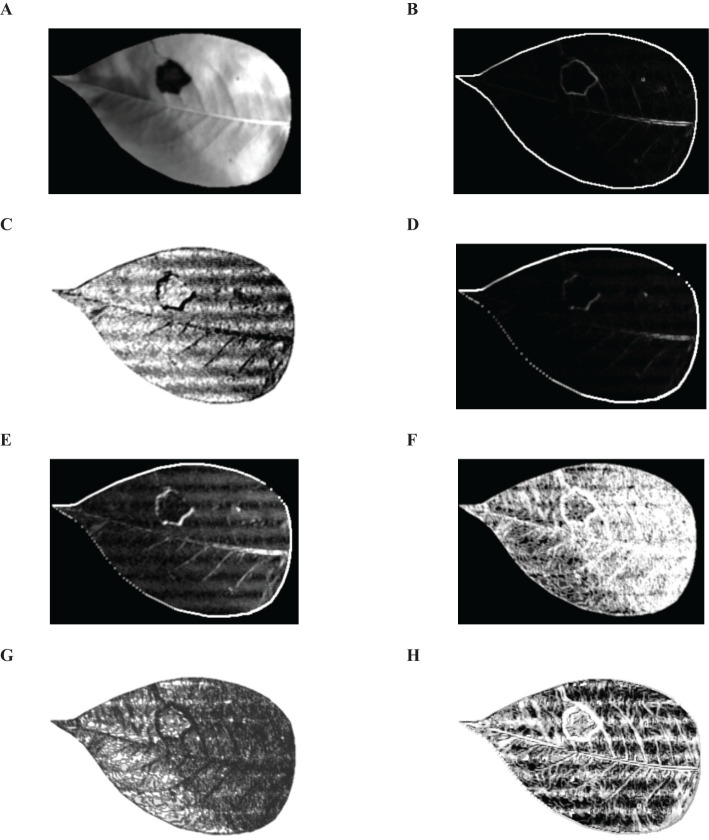
Eight common TFs. **(A)** Mean, **(B)** Variance, **(C)** Synergy, **(D)** Contrast, **(E)** Dissimilarity, **(F)** Entropy, **(G)** Second-order Moment, **(H)** Correlation.

In the experimental setup, a distance parameter of 1 was utilized for employing GLCM to extract TFs from images derived from the top 3 principal components. Analysis was conducted on four directional angles (0°, 45°, 90°, and 135°) with the results averaged. As illustrated in **Figure 8**, a total of 8 common TFs were extracted from the pear leaf images. And Mean, Dissimilarity, Entropy and Correlation were identified as particularly effective in accurately representing disease spot information. Consequently, 4 TFs were ultimately selected, yielding a total of 12 distinctive feature values that were used for building the classification model.

#### 3.3.2 Identification models with texture features

The SVM, ELM, and BPNN classification models were established based on the 12 distinctive features obtained above. **Table 5** described the testing set classification results of the identification models, with the accuracy rates of 65.83%, 71.11%, and 68.06%, respectively, for the classification models. Compared to models based on spectral features and VIs, the performance of the models was relatively lower. This could be attributed to the subtle changes in external leaf texture during the early stages of pear leaf anthracnose. However, the results indicated the feasibility of using TFs for early detection of pear leaf anthracnose.

**Table 5:** Classification results of identification models testing sets based on TFs features.

Input features	Number of variables	Model type	Accuracy (%)	Recall (%)	Precision (%)
TFs	12	SVM	65.83	68.23	65.87
		ELM	71.11	70.98	71.53
		BPNN	68.06	69.92	68.55

### 3.4 Identification models with multi-source features

Various combinations of feature wavelengths (OWs2), obtained through CARS extraction along with screened VIs and TFs, served as diverse inputs for constructing three classification models—SVM, ELM, and BPNN—for early detection of pear leaf anthracnose. It showed the results of detecting the testing set of multi-source feature fusion model in **Table 6**. Comparative analysis revealed enhanced identification accuracies across all models utilizing multi-source feature fusion compared to single-feature approaches; notably achieving over 90% accuracy when employing this method as a variable input—indicating that amalgamating multiple source features yields richer information pertinent to effective disease identification while enhancing overall model performance. Notably among these features was OWs2-VIs-TFs-BPNN which demonstrated superior efficacy in early detection of pear leaf anthracnose with an impressive accuracy rate reaching 98.61%.

**Table 6:** Classification results of identification models testing sets based on multi-source feature fusion.

Input features	Number of variables	Model type	Accuracy (%)	Recall (%)	Precision (%)
OWs2-VIs	12	SVM	92.50	91.94	92.92
		ELM	94.44	92.50	94.57
		BPNN	97.78	95.28	97.80
OWs2-TFs	20	SVM	94.17	95.28	94.37
		ELM	93.06	97.22	93.40
		BPNN	97.50	98.61	97.53
VIs-TFs	16	SVM	91.94	91.12	91.98
		ELM	92.50	93.54	92.65
		BPNN	95.28	94.78	95.42
OWs2 -VIs-TFs	24	SVM	95.28	94.92	95.33
		ELM	97.22	98.83	97.25
		BPNN	98.61	99.25	98.63

### 3.5 Visualization analysis of pear leaf anthracnose lesions

**Figure 9:**
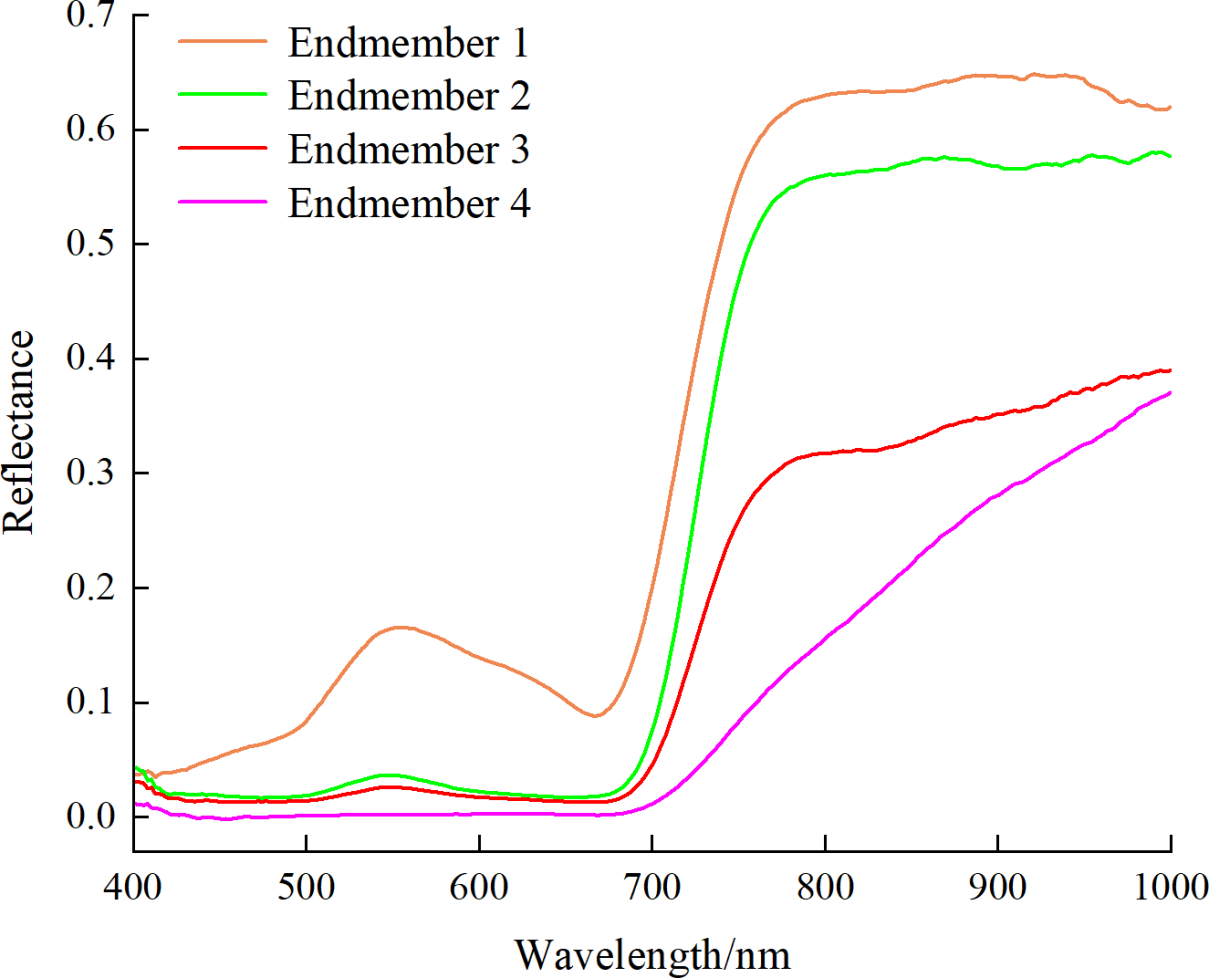
Different endmembers extracted by SMACC.

The hyperspectral images of healthy and diseased pear leaves from day 1 to day 5 were initially processed using SMACC to extract endmember spectra, followed by mixed pixel decomposition using SID to obtain SID images. Subsequently, disease tissue identification was conducted based on predefined reference spectra by comparing each pixel in the image with each endmember and assigning values. A total of 4 endmembers were extracted, and the colors in the SID image were set to be consistent with their corresponding endmembers. **Figure 9** displayed the 4 endmembers extracted based on SMACC. Endmember 1 (orange) represented the average spectrum of leaf veins, endmember 2 (green) signified the typical spectrum of healthy pear leaves, endmember 3 (red) represented the average spectrum of diseased leaf spots with lower reflectance, and endmember 4 (pink) corresponded to the average spectrum of dead leaf tissue, exhibiting reflectance curves no longer consistent with the typical spectral features of green plants.

**Figure 10:**
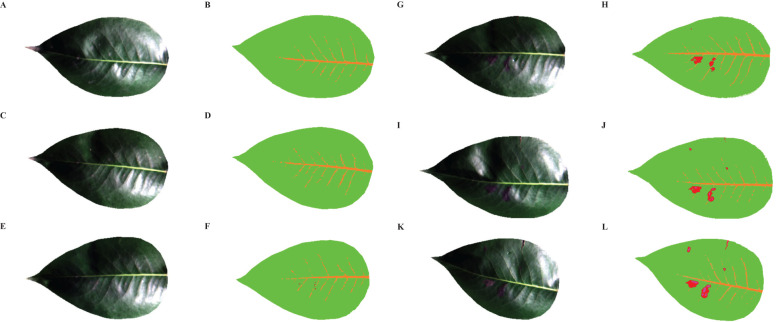
RGB images and SID images of healthy and infected pear leaves from 1 to 5 days. **(A)** RGB-Healthy, **(B)** SID-Healthy, **(C)** RGB-Infected Day 1, **(D)** SID-Infected Day 1, **(E)** RGB-Infected Day 2, **(F)** SID-Infected Day 2, **(G)** RGB-Infected Day 3, **(H)** SID-Infected Day 3, **(I)** RGB-Infected Day 4, **(J)** SID-Infected Day 4, **(K)** RGB-Infected Day 5, **(L)** RGB-Infected Day 5.

**Figure 10** illustrated the RGB and SID images of healthy and infected pear leaves from day 1 to 5. It could be observed that there were no discernible changes in the RGB images of the leaves from day 1 to 2. However, the SID images revealed the presence of minute lesions on the second day of infection. As time progresses, from day 3 to 5, symptoms of pear leaf anthracnose became increasingly prominent in the RGB images. Concurrently, in the corresponding SID images, the red lesions increased on the third day, pink necrotic leaves appeared on the fourth day, and by the fifth day, leaf infection reached its peak throughout the entire experimental phase. In conclusion, compared to the RGB images, the SID images effectively identified the symptoms of pear leaf anthracnose through changes in spectral characteristics rather than lesion size. As time progresses, the visualization of pear leaf anthracnose lesions became more effective.

## 4 Discussion

### 4.1 Importance of correct feature wavelength extraction

Hyperspectral data usually contain a lot of redundant information and highly correlated wavelength information, so it is very important to extract the feature wavelength (Siedliska et al., 2018; Lin et al., 2014). With the rapid development of computer technology, there are many methods to extract feature wavelength. However, the feature wavelengths used in different studies are quite different. Liu et al. (2022) used SPA algorithm to extract the feature wavelength when monitoring the anthracnose of infected pear leaves, and found that the important bands related to anthracnose were between 400-460nm. Wang et al. (2014) used the first-order differential to screen the spectral bands, and determined that the important bands related to tea anthracnose were between 680-780 nm. It can be seen that the feature wavelength bands of the same disease are very different, which may be due to different plant species or different extraction methods of feature wavelength. Therefore, in this study, in order to explore the influence of different feature methods on the detection of the same disease of the same plant species, SPA and CARS were used to extract the feature wavelength of pear leaf anthracnose. It was found that the feature bands extracted by SPA algorithm were concentrated in 400-560nm and 710-1000nm, while the feature bands extracted by CARS algorithm were concentrated in 710-1000nm. After modeling and analysis, it was found that CARS algorithm was superior to SPA algorithm, and the model constructed by CARS algorithm had higher recognition accuracy. Therefore, different extraction methods of feature wavelength have great influence on disease research and detection, and many methods are needed to study, and it is more important to choose the correct extraction method of feature wavelength.

### 4.2 Advantages of multi-source feature fusion in early detection of plant diseases

In previous studies, Zhu et al. (2019); Liang et al. (2023); Chen et al. (2021) and Gao et al. (2022) used single spectral feature to identify and detect rice sheath blight, sclerotinia sclerotiorum of rapeseed, verrucous mildew disease of agaricus bisporus and gray mold disease of tomato respectively, which could accurately identify the diseases in the obvious stage, but could not accurately identify them in the early stage. This was because in the early stage of the disease, the lesion was not obvious and the spectral features had not changed significantly. It was difficult to realize the early detection of diseases only by using a single spectral feature (Nguyen et al., 2021). However, in this study, besides spectral features, VIs and TFs were also considered. The early changes of pear leaf anthracnose were captured from many aspects, and the early accurate identification of pear leaf anthracnose was realized by building a model with multi-source feature fusion. This provided a theoretical basis for the early prevention and control of pear leaf diseases in the future. The fusion of multi-source features can better capture the subtle changes of disease symptoms in many aspects, thus providing greater possibilities for early and accurate detection of plant diseases (Zhang et al., 2020; Ren et al., 2020). However, at present, there are few studies on early detection of plant diseases by multi-source feature fusion, and most of them are based on single spectral feature. Therefore, many factors should be considered in the study of early diseases in the future, such as VIs, TFs, chlorophyll and nitrogen content (Lalit and Purwar, 2022). Striving to achieve accurate detection of diseases in the early stage, so that diseases can be prevented in time and economic losses can be reduced. I believe that with the continuous progress of related technologies, the detection method of multi-source feature fusion will play a more important role in future agricultural production.

### 4.3 Function of lesion visualization

In the early stage of plant diseases, the disease spots are not obvious, so some infection symptoms cannot be clearly identified only from RGB images, and early detection cannot be realized through image processing (Terentev et al., 2022). In contrast, hyperspectral images contain much more information than RGB images, and some effective methods can be used to visualize them, so that the location of lesions in the early stage of diseases can be tracked (Lowe et al., 2017). Zhou et al. (2019) and Li et al. (2016) respectively visualized the early stage of barley rice blast and citrus rot through SAM. However, when using SAM to extract different endmembers, we needed to manually classify different endmembers according to the differences in spectral characteristics. Because the difference of early spectral characteristics was not obvious, it leaded to some errors in manual classification. Eventually, their experimental results were unclear in the early stage of disease spot visualization. In this study, SMACC method could automatically capture spectral differences and extract typical endmembers according to the changes of spectral features caused by anthracnose of pear leaves. Then, the mixed pixel decomposition was carried out by SID, and the SID image was obtained. From the SID image, we could clearly see the shape, distribution and scope of the early stage lesions, and realize the visualization of the early stage lesions of pear leaf anthracnose. However, some preliminary experiments needed to be done in advance to determine the number of typical endmembers extracted before the formal experiment, which was helpful to better visualize the phenotype of the lesion. In a word, the effective visualization method of disease spots can accurately monitor the course of disease and provide scientific basis for the early prediction and prevention of subsequent plant diseases.

## 5 Conclusion

This study, based on HSI technology, utilized a combination of multi-source features and three machine learning models to enable early detection of pear leaf anthracnose. Healthy pear leaves and leaves infected with the disease for 1 to 5 days were used as the research subjects. Spectral reflectance was extracted from hyperspectral images in the range of 400 nm to 1000 nm. 12 and 8 feature wavelengths (OWs1 and OWs2) were respectively extracted using SPA and CARS. Then, 4 VIs were chosen via RF, and twelve TFs were extracted using GLCM after dimensionality reduction through PCA. Three types of features with different fusion methods were used as variable inputs to construct classification identification models (SVM, ELM, and BPNN). The results showed that the models constructed using multi-source feature fusion outperformed those using single features. Specifically, the OWs2-VIs-TFs-BPNN model exhibited the best performance in early detection of pear leaf anthracnose with an accuracy of 98.61%. Furthermore, SMACC and SID were employed to visualize the lesions on pear leaves at varying stages of anthracnose infection, enabling an intuitively monitoring of the disease progression. In summary, the early classification identification model and lesion visualization based on multi-source feature fusion provide scientific support for the early detection and precise treatment of pear leaf anthracnose. In future research, it would be valuable to explore the use of HSI technology combined with deep learning for the early detection of pear leaf diseases, and to apply these techniques in field environments.

## Data Availability

The raw data supporting the conclusions of this article will be made available by the authors, without undue reservation.
